# Photocurable Coatings Based on Bio-Renewable Oligomers and Monomers

**DOI:** 10.3390/ma14247731

**Published:** 2021-12-15

**Authors:** Paulina Bednarczyk, Małgorzata Nowak, Karolina Mozelewska, Zbigniew Czech

**Affiliations:** Department of Organic Chemical Technology and Polymer Materials, Faculty of Chemical Technology and Engineering, West Pomeranian University of Technology in Szczecin, Pułaskiego Ave. 10, 70-322 Szczecin, Poland; nowak.malgorzata@zut.edu.pl (M.N.); karolina_mozelewska@zut.edu.pl (K.M.); psa_czech@wp.pl (Z.C.)

**Keywords:** acrylated epoxidized soybean oil (AESO), bio-based coatings, photopolymerization, kinetic

## Abstract

Due to long-term problems related to environmental protection, economic aspects, and waste management in the chemical industry, it is justified to develop renewable polymers as an alternative to synthetic polymers. Two kinds of acrylic bio-renewable components were used for the modification of acrylated epoxidized soybean oil (AESO). The bio-based compositions used as photocurable binders to obtain the photocurable coatings with satisfactory properties and high bio content were then prepared. The kinetic of curing reaction of the oligomers and monomers towards radical photopolymerization and the properties of the cured coatings were fully investigated; the results are discussed in relation with the compounds’ structures. Important information about how to design and obtain renewable photocurable coatings with satisfactory properties was provided in this study. In this study, AESO resin was modified with renewable oligomer or (math)acrylate monomer to increase the reactivity and reduce the viscosity of the photoreactive system in order to obtain renewable and viable alternatives to petroleum-based polymeric materials with perfect film-forming properties. It turned out that both photopolymerization rate and hardness of cured coatings were increased significantly with the addition of modifiers; the use of a thiol modifier and change of the photoinitiator concentration allowed to improve the adhesion, hardness, and control of the photo-curing process.

## 1. Introduction

The use of UV technology for curing polymers is a fast way to change liquid, reactive materials into solid materials with good properties, for applications such as coatings, adhesives, inks, and dental materials [1,2,3]. The method of curing polymers using UV radiation is considered green technology, because it is characterized by low VOC emissions, high efficiency, and low energy consumption [4,5,6]. It is desirable to combine bio-renewable materials and “green” UV-curable technologies. Due to the presence of increasingly strict environmental regulations, which are particularly relevant to the chemical industry, this combination provides a “green + green” solution [3,5,7,8,9,10].

In recent years, there has been an increased demand for the replacement of petroleum-derived polymeric materials with bio-renewable polymers [5,7,8], due to the growing concern for the environment as well as forecasts of the shortage of crude oil resources [5,9]. Polymers obtained from renewable raw materials are already applied in packaging, electronics, bio-medical and hygiene products, cosmetics, agriculture, other consumer goods, and have even been tried in optical 3D printing [11,12,13,14]. Natural resources such as vegetable oils, fatty acids, or lactic acids have been found to be particularly widely attractive for scientists because they can be molecular-engineered into renewable polymers in a way similar to many petroleum chemicals. [15]. Vegetable oils are an interesting renewable resource as they are abundant, cheap, and easily modifiable, because they contain several highly reactive sites [10,16,17,18]. Natural plant oils are applied to prepare bio-based materials curing by UV radiation via various chemical modifications, e.g., by acrylation and epoxidation. Both the fatty unsaturation peroxide oxidation and acrylic acid ring open epoxy process lead to acrylated or epoxidized plant oils for free radical or cationic UV curable materials [19,20,21,22]. Recently, acrylated sunflower oil was prepared by a new method that involves the reaction of “ene” with singlet oxygen [23]. In addition, vegetable oils can be used to produce thiols and enes by a ring opening epoxidized oils and use in UV curable materials by thiol-ene chemistry [3,24,25,26]. A norbornyl epoxidized linseed oil was synthesized by Chen et al. [27] via a Diels–Alder reaction of linseed oil with cyclopenta-diene, which is characterized by a high UV curing rate compared to epoxidized linseed oil due to the higher ring strain of the incorporated norbornyl groups.

Soybean oil (SBO) is regarded as abundant and low-cost feedstock; therefore, it is one of the most widely used vegetable oils in coatings. Soybean oil is currently mostly used for food applications. Refined SBO is composed of 99% triglycerides with an average molecular weight approximately 871 g/mol, with an average functionality of 4.6 carbon–carbon double bonds per triglyceride, and including eight different fatty acids ranging in length from 14 to 22 carbons long [22]. Materials directly obtained by polymerization of SBO are viscous oils or weak rubbery films that possess little utility as coating films [28]. Therefore, it is necessary to modify the SBO with functional molecules (i.e., introduction of more reactive functional groups), especially for it to be useful for film-forming applications, because the double bonds present in the soybean oil particles characterized by low reactivity and the triglyceride fatty acid structure are too flexible. Hence, soybean oil-derived chemicals, especially the acrylated epoxidized soybean oil (AESO) synthesized by Baipai et al., is an important bio-renewable resin for the development of the coatings industry [6,22,29,30,31,32]. In addition, AESO can also be used with numerous applications in the preparation of paints, varnishes, and related materials [33].

The cured coating based on AESO alone does not have properties of rigidity and strength required for this type of application, as compared to their petrochemical-based counterparts. The main reasons for the inferior properties of the coatings can be the lower reactivity for the mid-chain acrylates which leads to a low crosslinking degree or a soft fatty acid triglyceride backbone [34,35,36]. Thus, further development of these bio-based UV-curable materials aims at increasing their T_g_ and performance. In order to improve the performance of coatings based on AESO, some crosslinking agents characterized by rigid molecular structure or bio-based resins with a custom structure have to be incorporated into AESO. One promising agent for this purpose is found in the growing field of bio-renewable acrylic oligomers and monomers [4]. With the advantages of high cure speed, low viscosity, reduced energy consumption, and absence of VOC emission [29,31], acrylic oligomers or monomers are expected to produce UV-curable films with better crosslink density and thus significantly improved coating properties. AESO was also modified to increase the content of reactive unsaturated bonds, thereby increasing the cross-link density of biopolymers by reacting with maleic acid. In the reaction of the isocyanate prepolymer, a series of UV-curable oligomers with good film-forming properties were obtained, which were based on a vegetable oil containing allyl, acrylate, and vinyl groups [37]. It was also possible to increase the Tg of AESO by incorporating stiff cyclic rings into the AESO backbone by reacting with cyclohexane dicarboxylic acid [38]. AESO has also been UV-cured with “click” chemistry, such as thiol-ene by reactions with various petroleum-based thiols [39,40,41].

The possibility of using renewable raw materials to obtain film-forming materials has recently become the basis for increasingly devoting attention and efforts of academic and industrial researchers to the investigation and synthesis of “green” raw materials for coatings. Thus, in this paper, we report the modification of AESO resin with different acrylated bio-renewable oligomers and monomers allowing the obtainment of a hybrid biopolymer by a photochemically initiated reaction. Subsequently, photoreactive coating compositions were prepared from the obtained modification of UA with radical photoinitiator and later cured using UV radiation. The samples were investigated in terms of the course of the photopolymerization process and the cured coatings properties. Consequently, the results are discussed in relation with the components’ structures.

## 2. Materials and Methods

### 2.1. Materials

The following raw materials were used for the studies: epoxidized soybean oil acrylate resin (Ebecryl 5848; 62% biorenewable content, AESO) and bio-based diacrylate oligomer (Ebecryl 5850; 56% biorenewable content; BioDA) were provided from Allnex Resins, Germany GmbH and used without purification; isobornyl methacrylate (Sarbio 6105; 70%; BioMA) was purchased from Arkema; mercapto-modified polyester acrylate resin (Ebecryl LED 02; T) was purchased from Allnex Resins, Germany GmbH; and (2,4,6-trimethylbenzoyl)diphenylphosphine oxide used as photoinitiator (Omnirad TPO-L; PI) was provided by IGM resins.

### 2.2. Preparation of Coatings

**Figure 1:**
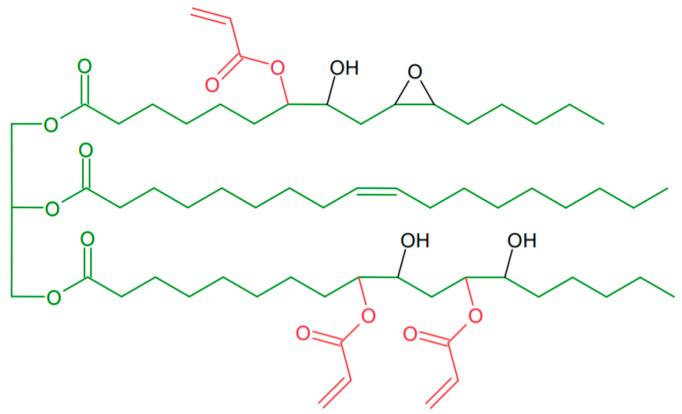
Theoretical structure of epoxidized soybean oil acrylate (AESO).

The coating compositions were formulated using epoxidized soya oil acrylate (AESO) as main component. Figure 1 shows the theoretical chemical structure of AESO. The predetermined AESO, Bio-DA, or Bio-MA, T, and various amount of PI (on the basis of the total weight of photoreactive components) were mixed together until homogeneous formulations were obtained. Then, the curing mixture was applied by means of a gap applicator (120 µm) to the glass substrate. The polymeric film was cured under a UV lamp (Aktiprint-mini 18-2, type: UN50029, Technigraf GmbH) with an intensity of 200 mW/cm^2^ at room temperature to dryness. Table 1 shows the composition of the tested formulations.

### 2.3. Characteristics of the Photopolymerization Process

The UV-curing process of coatings based on AESO was monitored using photo-DSC apparatus (Q100, TA Instruments, New Castle, DE, USA) equipped with a UV light emitter Omnicure S2000 (100 mW/cm^2^, 280–480 nm, Excelitas Technologies, Waltham, MA, USA), isothetmally (25 °C) for 15 min.

The Fourier-transformed infrared (FTIR) spectroscopy in the attenuated total reflectance mode (ATR) was measured on Nicolet iS5 instrument (Thermo Electron Corporation). For each sample, the average of sixteen scans was in the range of 4000–400 cm^−1^. The infrared spectra were used to characterize the received AESO modification, allowing the obtainment of a hybrid polymer by photochemically initiated reaction. In addition, the series real-time IR (RT-IR) was used to determine the conversion of unsaturated chemical bonds. More importantly, this spectroscopic technique permits in situ monitoring of the chemical processes via mimicking the disappearance of the characteristic bands of the reactive compounds subjected to UV exposure. The thin photoreactive polymer films (0.5 in thickness) were placed at the measuring site of a FTIR spectrometer and exposed to a source of UV radiation (mercury UV lamp, 36 W, 280–400 nm, 10 mW/cm^2^) and an IR analysis light beam. The absorbance change of the acrylate double bond (C = C) peak area was correlated to the extent of polymerization. The degree of conversion (DC) can be expressed by the following relations: DC (%) = (A_0_ − A_t_)·100/A_0_, where A_0_ is the initial peak area before irradiation and At is the peak area at time t. The photopolymerization rate (Rp) was calculated by the following relations: Rp = dDC/dt, where t is the time of irradiation.

### 2.4. Properties of Cured Coatings

To evaluate the properties of the cured coatings, the following tests were performed: tack-free time, pendulum hardness test, adhesion, gloss, and yellowness index. According to the ISO 9117 standard, the tack-free time was tested in order to evaluate the surface cure time. It is the time at which the coating is deemed to be properly adhered to and achieves the final technical parameters. According to ASTM D 4366 standard, the hardness of cured coatings was tested using König pendulum hardness tester with respect to pendulum oscillation times on the coatings on the glass substrate. According to ISO 4624 standard, the adhesion of the cured coatings to the glass substrate was measured by the use of the pull-off adhesion tester. According to ASTM D523 standard, the gloss of the cured coatings was measured by spectrometer GLS (SADT Development Technology Co. Ltd., Beijing, China). According to ASTM E313 standard, the yellowness index of the cured coatings was measured using a precision colorimeter NH-145 (3NH Technology Co. Ltd., Shenzhen, China).

## 3. Results and Discussion

### 3.1. Modification of AESO-Based Coatings

The acrylated epoxidized soybean oil (AESO) may be a crucial industrial photoreactive bio-based compound, on condition that it is modified to introduce raw materials improving its properties. The AESO should not be used alone because of its poor thermal and mechanical properties [42]. In this study, AESO resin was modified with renewable oligomer or (math)acrylate monomer to increase the reactivity and reduce the viscosity of the photoreactive system in order to obtain renewable and viable alternatives to petroleum-based polymeric materials with perfect film-forming properties. A typical photo-curing system contains three basic components: (1) the acrylated functionalized oligomer which constitutes the backbone of the polymer network; (2) the acrylated monomer which acts as a reactive diluent; and (3) a photoinitiator which can generate free radicals by photolysis. The photoinitiator plays a key role in the polymerization process, and it determines both the rate of polymerization and the cure depth. Therefore, the research also presents AESO-based systems with a different amount of radical photoinitiator being tested for photopolymerization behavior and influence on the properties of cured coatings. Additionally, mercapto-modified polyester acrylate resin was also introduced into the AESO-based system to increase reaction speed, cure coatings with mild reaction conditions, and reduce the effects of oxygen inhibition. Therefore, the main aim of this work was to study the photopolymerization kinetics of systems based on AESO modified with renewable oligomers and acrylate monomers, as well as the study of basic methods of photoreactive system modification in order to obtain coatings under mild conditions with good properties.

### 3.2. Photocuring Process of Modified AESO-Based Coatings

The interest of scientists and the industry in photoreactive resins synthesized from renewable sources has been growing for several years. These were achieved by introducing acid functionality and C = C groups onto triglyceride molecules, which undergo free radical polymerization. These types of photoreactive resins have environmental and economic advantages and are an attractive alternative over petroleum-based resins. AESO resin used in research as a starting material has an average of 3.4 acrylate groups per triglyceride. First, the AESO-based composition was characterized for photopolymerization behavior and compared with a difunctional acrylate oligomer which was also obtained from renewable sources.

**Figure 2:**
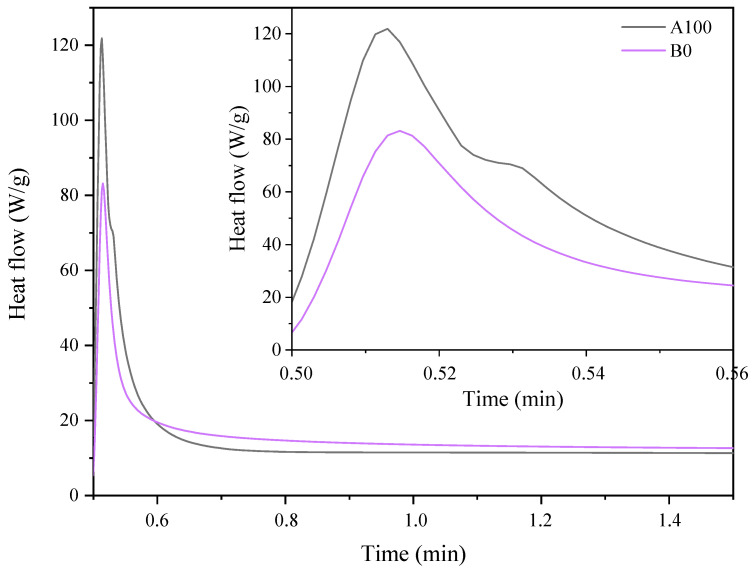
Photo-DSC exotherms for the photopolymerization of AESO composition (A100) and bio-based diacrylate oligomer composition (B0).

**Figure 3:**
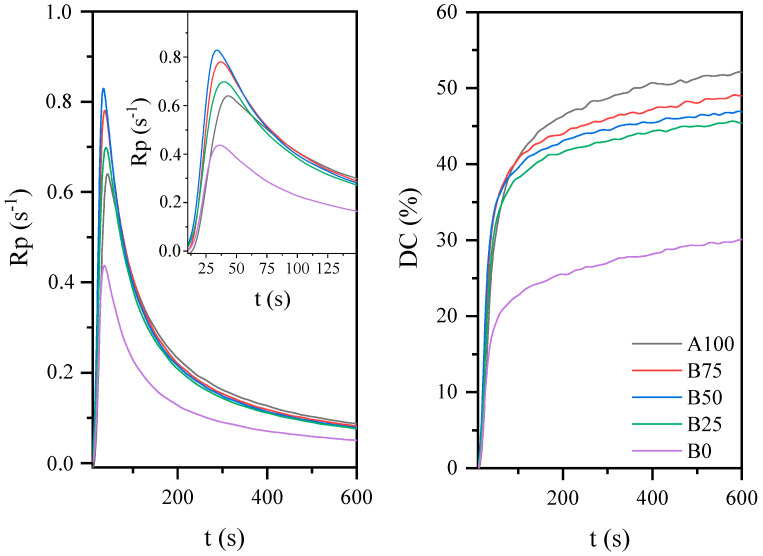
The effect of acrylate oligomer concentration on the photopolymerization process of coatings based on the AESO.

Figure 2 show the photo-DSC exotherms for the photopolymerization of the investigated UV-curable systems. Both systems have a similar course of the curves showing the photopolymerization process, except that the system based on AESO has a higher exotherm than the other system. This may be related to higher polymerization reactivity of the first system compared to the second system due to the presence of more unsaturations in the oligomer molecule, i.e., its functionality. The acrylated epoxidized soybean oil has three acrylate groups in the molecule, while BioDA has two groups. The photopolymerization behavior of these systems was also observed by IR spectra analyses (Figure 3). The studies also included AESO:BioDA compositions in various weight ratios. The system based on BioDA polymerizes the slowest, probably due to the lower reactivity associated with the functionality of this biooligomer. However, it appears that the AESO:BioDA mixtures polymerize faster than the AESO or BioDA systems, in particular in a 1:1 weight ratio. This is probably due to a decrease in the viscosity of the AESO based system (AESO viscosity: ≅20,000 mPa·s; BioDA viscosity: 5000 mPa·s). The reduction of the system viscosity causes facilitation of carbon chain movements and the availability of photoreaction.

As a result, the combination of the properties of natural and synthetic polymers can obtain “hybrid” coatings. Due to the fact that there is a low availability of photoreactive bio-renewable resins on the market, it is worthwhile to modify them in order to obtain the desired properties. These materials can combine the low cost of natural source resins with the high performance of synthetic resins. Their properties lie between those displayed by the all-synthetic and all-natural resins.

**Figure 4:**
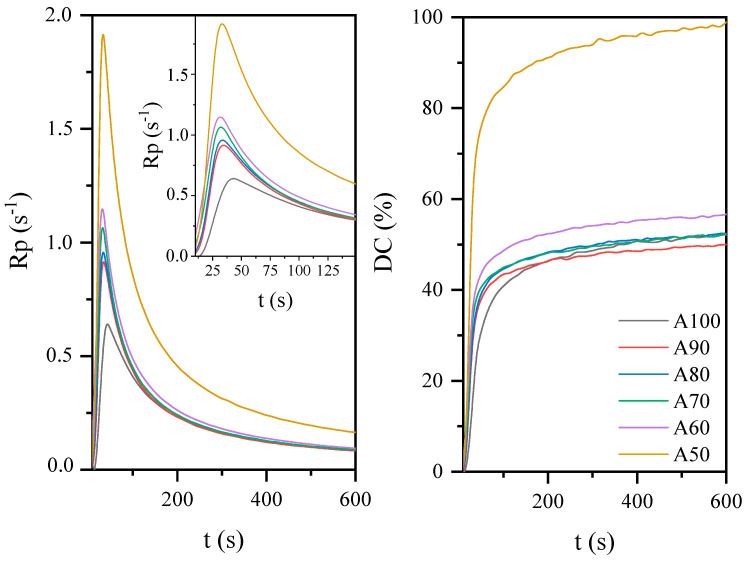
The effect of acrylate monomer concentration on the photopolymerization process of coatings based on the AESO.

AESO can also be blended with a reactive diluents, such as acrylate monomers, to improve its processability and control the photocuring process and cured coating properties to reach a range acceptable for the desired applications. By varying the amount of acrylate monomer, it is possible to produce coatings with different photopolymerization rate and conversion. In the research, isobornyl methacrylate was used as a reactive diluent and a renewable acrylic monomer, simultaneously. Terpenes prove to be a wide and diverse class of renewable organic compounds that include a large structural and functional variety, including photoreactive groups [43,44]. As shown in Figure 4, the addition of IBOMA increased the rate of polymerization and the degree of unsaturated bonds. As with the AESO:BioDA compositions, this may be associated with a reduction in the viscosity of the systems. Typically, oligomers are too viscous to be applied to the substrate by conventional methods and are therefore diluted with acrylate monomers to achieve the desired viscosity. The acrylate monomers used to obtain photocurable coatings are most often compounds with a molecular weight of 150 to 500 g/mol and usually contain from 1 to 4 acrylate or methacrylate functional groups per molecule. Unlike solvents, acrylate monomers copolymerize with acrylate oligomers and become an integral part of the cured coating and give it specific properties. Monomers are used as low-molecular-weight reactive diluents which not only regulate the viscosity of the system, but also affect the polymerization rate and polymer cross-link density (e.g., by increasing the concentration of double bonds in the system) [45]. Thus, the viscosity has a crucial effect on reaction kinetics and is related to the radical diffusion coefficient, which affects the control of initiation and propagation stages. Zhang et al. showed that the final conversion of the double bonds was related to the concentration of acrylate groups and the initial viscosity of the system [46,47,48].

**Figure 5:**
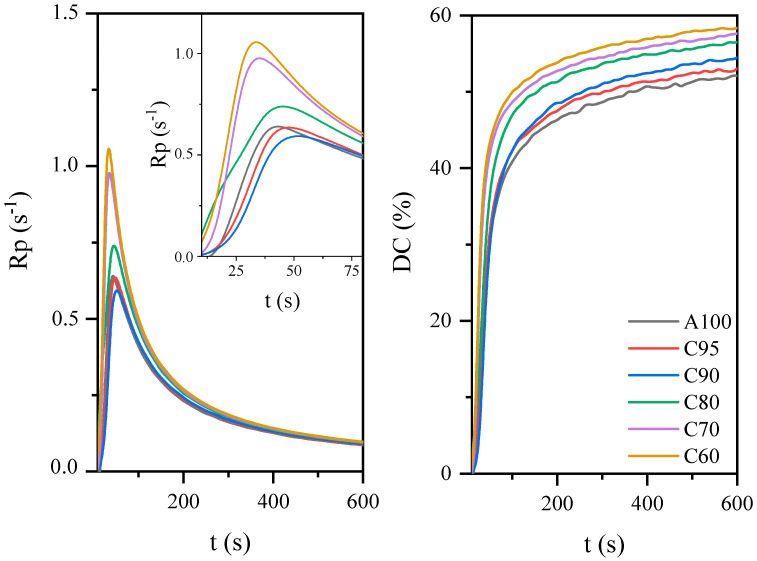
The effect of the thiol modifier on the photopolymerization process of coatings based on the AESO.

The polymer properties can also be controlled by changing the functionality of the acrylated triglyceride or modifying photoreactive compositions with various types of photoreactive additives. Consequently, a range of new properties and, therefore, new applications can be found. As a result of AESO acrylating, residual amounts of unreacted epoxy rings as well as newly formed hydroxyl groups may be present in the molecule structure, both of which can be used to further modify the triglyceride by reaction with a number of chemical species (e.g., diacids, diamines, anhydrides, and isocyanates). In this research work, the modification of the composition based on AESO with mercapto-modified polyester acrylate resin and changes of the photoinitiator concentration were used, mainly in order to accelerate the photopolymerization reaction while maintaining the properties of cured coatings. Recently, light-induced thiol-ene “click” reactions have been considered an attractive method of producing high-quality coatings [49], which are distinguished by reaction speed, resistance to oxygen inhibition, and low polymerization shrinkage [50]. Given the advantages of vegetable oils, click reactions, and UV curing, the preparation of vegetable oil-based thiol-ene resins for coatings was chosen. In this study, the curing kinetics of acrylated epoxidized soybean oil as biobased resin for thiol-ene and UV curing with commercially available thiols is presented. As shown in Figure 5, the addition of thiol increases the photopolymerization rate and the conversion of unsaturated bonds. However, as reported in literature, in thiol-ene click reactions of acrylates, not only step-growth thiol-ene copolymerization, but also chain-growth homopolymerization of acrylate can occur; consequently, thiols remain partially unreacted [51]. Unreacted groups can be further polymerized or can be used to obtain a partially cross-linked polymer in a controlled manner. Such properties can help to increase the density of the polymer network, glass transition temperature, or improve the polymer properties. Thiol-ene cross-linked polymers have been used in coatings, adhesives, biomedical, and electronic packaging materials [52]. Thus, this type of modification may contribute to the replacement of synthetic photoreactive polymers by renewable polymers.

**Figure 6:**
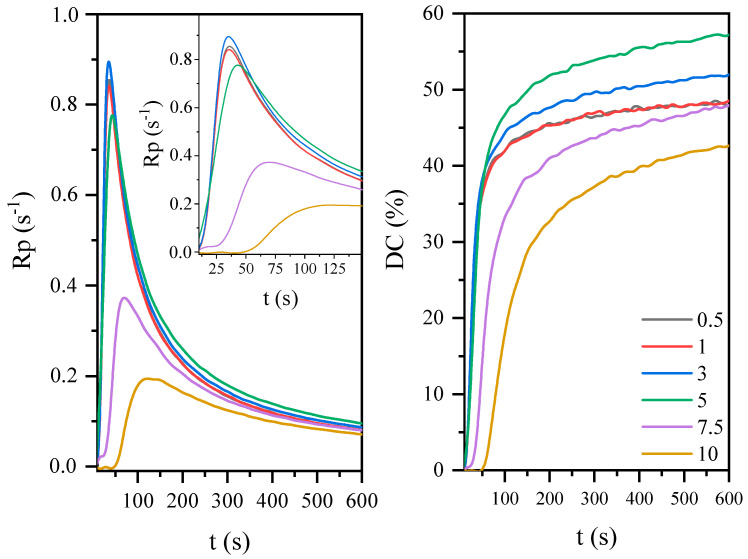
The effect of photoinitiator concentration on the photopolymerization process of coatings based on the AESO.

As is well known, the kinetics of the curing process of photoreactive coatings is also influenced by the type and concentration of the initiator. By changing the concentration of the photoinitiator in the system, the curing process rate can be controlled (Figure 6). As it turns out, there is a maximum concentration that determines the fast curing process (3 wt.% or 1–5 wt.%). Above and below this concentration, the process runs slower, obtaining a lower conversion degree of unsaturated bonds, which also affects the properties of the cured coatings. Photoinitiators absorb radiation and initiate the polymerization reaction. Therefore, increasing the concentration of initiator can induce a quick and high generation of free radicals, yielding the production of a faster polymerization kinetics reaction and higher degree of conversion. However, studies have shown that there is an ideal level for the increase of photoinitiator concentration and above this level, the increase in photoinitiator concentration does not benefit the final degree of conversion. This may be due to the presence of more radicals in the early stage of photopolymerization and thus increasing polymer network density, stiffening the reaction environment, and hindering the mobility of unreacted macroraders that terminate prematurely, which is reflected in the low conversion of unsaturated bonds.

### 3.3. Properties of Cured Coatings

Due to the fact that soybean oil is considered an abundant and low-cost feedstock, the oligomers based on the soybean oil are one of the most widely used renewable photoreactive components used in coatings. Moreover, they are easily accessible commercially. However, defect of these bio-based UV curable coatings is the inherent “softness” owing to the presence of the long fatty chains, which results in lower materials T_g_ that subsequently limits the plant oil-based materials’ applications as durable materials. The hardness of the cured coating obtained solely on AESO was low and amounted to 49 (Table 2). Hence, it is advantageous to modify this type of resin in order to increase the crosslink density and incorporation of more rigid structures onto the polymer network. The use of renewable oligomers with shorter carbon chains and the addition of monomer increases the hardness of the obtained coatings several times, which gives the opportunity of their practical application. In addition, the use of the monomer also resulted in an increase in adhesion and gloss and a reduction in yellowness. Series C deals with the use of thiol-ene chemistry. The importance of thiol molecules in renewable photoreactive systems has recently generated significant academic interest in the impact of this reaction, especially since it can lead to shortened tack-free time, reducing the adverse effects of oxygen inhibition and increasing adhesion. The properties of the cured coatings can also change using a different amount of photoinitiator. Increasing its concentration causes an increase in hardness and gloss, but it is also associated with a reduction in adhesion and yellowing of the coatings.

## 4. Conclusions

As we live in a time of heightening environmental issues, materials science is required to prepare sustainable polymers through green chemistry ideals. The studies showed that it is possible to control the curing process and the properties of cured coatings obtained from renewable photoreactive sources by simply modifying the photoreactive renewable resins without having to reduce biobased carbon content. In this work, four series of UV curable soybean hybrid coatings were studied based on renewable oligomers or monomers, various amount of photoinitiator, and thiol-acrylate system. Both photopolymerization rate and hardness of cured coatings were increased significantly with the addition of modifiers. These parameters were mainly influenced by the use of bio-renewable substrates with a lower viscosity than AESO, i.e., oligomer (BioDA) and monomer (BioMA). Hence, the AESO:BioDA mixtures polymerized faster than the AESO or BioDA systems, in particular in a 1:1 weight ratio, and the dilution of AESO with monomer (50 wt.%) resulted in an approximately threefold increase in the polymerization rate and almost 100 percent conversion of unsaturated bonds. Thus, the reduction of the system viscosity probably caused facilitation of carbon chain movements and the availability of photoreaction. The increase in the rate of photopolymerization and C = C conversion was also obtained using a thiol modifier. In this case, an improvement in the adhesion of the coatings was also achieved. The research also selected the concentration of photoinitiator, which determined high conversion (3 wt.% or 1–5 wt.%) and influenced the properties of the cured coatings. This type of material can be very promising for the coating industry application, in particular to the development of advanced bio-based UV-curable materials from any type of unsaturated plant oils and obtaining cured polymer in a short time, using renewable raw materials and “green” methods.

## Figures and Tables

**Table 1:** Composition based on UV-curable AESO modified with biocomponents (A series with acrylate monomer and B series with acrylate oligomer) and other components (C series with mercapto-modified polyester acrylate resin and D series with various amount of photoinitiator).

Samples		Weight Ratio (wt%)				Biobased Content (wt%)
	AESO	BioDA	BioMA	T	PI	
A-100	100	-	-	-	3	56
A-90	90	-	10	-	3	57.4
A-80	80	-	20	-	3	58.8
A-70	70	-	30	-	3	60.2
A-60	60	-	40	-	3	61.6
A-50	50	-	50	-	3	63
B-75	75	25	-	-	3	57.5
B-50	50	50	-	-	3	59
B-25	25	75	-	-	3	60.5
B-0	-	100	-	-	3	62
C-95	95	-	-	5	3	53.2
C-90	90	-	-	10	3	50.4
C-80	80	-	-	20	3	44.8
C-70	70	-	-	30	3	39.2
C-60	60	-	-	40	3	33.6
D-0.5	80	20	-	-	0.5	58.8
D-1	80	20	-	-	1	58.8
D-3	80	20	-	-	3	58.8
D-5	80	20	-	-	5	58.8
D-7.5	80	20	-	-	7.5	58.8
D-10	80	20	-	-	10	58.8

**Table 2:** Properties of cured coatings based on the AESO.

Sample	Tack-Free Time (s)	Pendulum Hardness	Crosscut Adhesion	Gloss (GU)	Yellowing Index
A-100	-	49	4	122	5.00
A-90	-	66	2	132	4.66
A-80	-	100	2	134	4.47
A-70	-	128	1	135	4.35
A-60	-	130	1	138	4.12
A-50	9	133	1	140	4.35
B-75	-	97	2	105	4.67
B-50	-	134	3	103	4.42
B-25	-	205	3	97	422
B-0	9	260	3	91	3.95
C-95	-	52	2	113	4.63
C-90	-	52	2	111	455
C-80	-	42	2	106	4.73
C-70	-	36	1	103	4.61
C-60	9	36	0	101	4.80
D-0.5	-	53	1	38	4.40
D-1	-	74	2	67	4.44
D-3	-	100	2	134	4.47
D-5	-	104	2	136	4.57
D-7.5	-	116	3	138	4.87
D-10	-	118	4	140	5.37

## Data Availability

Not applicable.
